# A Metabonomics Profiling Study on Phlegm Syndrome and Blood-Stasis Syndrome in Coronary Heart Disease Patients Using Liquid Chromatography/Quadrupole Time-of-Flight Mass Spectrometry

**DOI:** 10.1155/2014/385102

**Published:** 2014-07-20

**Authors:** Linlin Zhao, Ling Wan, Xinjian Qiu, Ruomeng Li, Shimi Liu, Dongsheng Wang

**Affiliations:** Integrated Traditional Chinese and Western Medicine, Xiangya Hospital, Central south University, Changsha 410008, China

## Abstract

A metabonomics approach based on liquid chromatography/quadrupole time-of-flight mass spectrometry (LC-Q-TOF/MS) was utilized to obtain potential biomarkers of coronary heart disease (CHD) patients and investigate the ZHENG types differentiation in CHD patients. The plasma samples of 20 CHD patients with phlegm syndrome, 20 CHD patients with blood-stasis syndrome, and 16 healthy volunteers were collected in the study. 26 potential biomarkers were identified in the plasma of CHD patients and 19 differential metabolites contributed to the discrimination of phlegm syndrome and blood-stasis syndrome in CHD patients (VIP > 1.5; *P* < 0.05) which mainly involved purine metabolism, pyrimidine metabolism, amino acid metabolism, steroid biosynthesis, and arachidonic acid metabolism. This study demonstrated that metabonomics approach based on LC-MS was useful for studying pathologic changes of CHD patients and interpreting the differentiation of ZHENG types (phlegm and blood-stasis syndrome) in traditional Chinese medicine (TCM).

## 1. Introduction 

Coronary heart disease (CHD) usually associates with high morbidity and mortality; the incidence of CHD increases year by year and shows younger tendency [1]. Coronary angiography has been the “golden standard” for the diagnosis of CHD [2, 3]. CHD is considered a “thoracic obstruction” (Xiongbi in Mandarin) in traditional Chinese medicine (TCM) [3, 4]. Blood-stasis syndrome and phlegm syndrome are identified as the main ZHENG types (TCM syndrome or pattern) in patients with CHD [5]. ZHENG types are essential element of TCM theory and classified according to symptoms; different therapies result from different ZHENG types. The biological basis in microcosmic level of ZHENG types has been a study hot spot on standardization and modernization of TCM [6]. Metabonomics is defined as “the quantitative measurement of the dynamic multiparametric response of a living system to pathophysiological stimuli or genetic modification” [7, 8]. It provide “an accurate, noninvasive and rapid diagnosis of coronary heart disease” [9]. Moreover its systemic, holistic, and dynamic characteristics are coincident with TCM theory, and it has been used to study ZHENG types differentiation and curative effects of Chinese medicine [10–13]. Analytical platforms of metabonomics include ^1^H-NMR, FT-IR, GC-MS, GC × GC-MS, LC-MS, and CE-MS [14–16]. Considering the complexity of the metabolites, none of the analytical platforms can be used alone to find all of the metabolites and a combination of methods is necessary to obtain as many metabolites as possible [16, 17]. In our study, CHD patients with phlegm and blood-stasis syndrome were selected applying liquid chromatography quadrupole time-of-flight mass spectrometry (LC-QTOF/MS) to study the underlying essence of the disease (CHD) and ZHENG types (phlegm syndrome and blood-stasis syndrome).

## 2. Method

### 2.1. Subjects

40 CHD patients (20 with phlegm syndrome and 20 with blood-stasis syndrome), confirmed by coronary angiography, came from Xiangya Hospital affiliated to Central South University (Hunan, China) from March 1, 2012, to June 30, 2013. Diagnosis standard of CHD based on “nomenclature and diagnosis criteria of ischemic heart disease” is established by the Joint International Society and Federation of Cardiology/World Health Organization Task Force on Standardization of Clinical Nomenclature [18]. The ZHENG types were identified by three chief physicians, according to “criteria for TCM syndrome differentiation of patients with coronary heart disease” [19].

16 age and sex matched healthy volunteers were enrolled from medical examination center in the same hospital. A questionnaire was used to survey lifestyle and drugs intake within a week. The clinical data of each subject were shown in the Supplementary Material available online at http://dx.doi.org/10.1155/2014/385102.

Excluded cases contained patients who suffered from diabetic cardiomyopathy, hyperthyroid heart disease, hypertensive heart disease, pulmonary heart disease, anemic heart disease, systemic scleroderma heart disease, inborn coronary abnormity, and rheumatic heart disease, who suffered from severe hypertension, malignant tumor, renal failure, thyroid disease, diabetes mellitus, and pulmonary infection, who suffered from infectious diseases, such as hepatitis and tuberculosis, and who suffered from invigorative system disease and women in pregnant or in lactation.

The study was approved by the hospital ethics committee and all subjects gave written informed consent.

### 2.2. Chemicals

HPLC-grade acetonitrile, methanol, formic acid, and ammonium formate were obtained from Merck & Co Inc. (NJ, USA). Water was distilled thrice from silica glass equipment before use.

### 2.3. Samples Collection and Preparation

5 mL venous plasma samples were collected into ethylenediaminetetraacetic acid (EDTA) vacutainer tubes in the morning after 12 h of overnight fasting and then centrifuged at 10000 rpm for 10 min at 4°C; the supernatant was frozen at −80°C until analysis.

The samples at room temperature thawed for 15 min and vortexed for 5 s before use. Each 100 *μ*L of plasma was diluted with 300 *μ*L methanol; after being vortexed for 30 s, the mixture was stored at 4°C for 10 min and then centrifuged at 12000 rpm and 4°C for 15 min. The supernatant was extracted to autosampler.

### 2.4. LC-MS Instrumentation and Analytical Condition

The assay was performed on LC-Q/TOF-MS using Agilent 1290 Infinity LC coupled with an electrospray ionization (ESI) source and Agilent 6530Q-TOF mass spectrometer (Agilent Technologies, Palo Alto, CA, USA). The LC separation was carried out on a ZORBAX RRHD SB-C_18_ column (2.1 × 100 mm, 1.8 *μ*m, Agilent) with the column temperature maintained at 40°C. The mobile phases consisted of ultrapure water (A) and acetonitrile (B) both containing 0.1% (v/v) formic acid; the gradient program was shown in Table 1. The injected volume of sample was 4 *μ*L and temperature of autosampler was 4°C.

Nitrogen was used as both nebuliser gas and cone gas. Detection mode of flight tube was type V. The mass spectrometric data was collected in both positive and negative modes with the following parameters: capillary voltage 4 kV (positive mode) and 3.5 kV (negative mode), sampling cone voltage 35 kV (positive mode) and 50 kV (negative mode), source temperature 100°C, desolvation temperature 350°C (positive mode) and 300°C (negative mode), cone gas flow rate 50 L/h, desolvation gas flow rate 600 L/h (positive mode) and 700 L/h (negative mode), extraction cone voltage 4 V, and full scan mode scanning from* m*/*z* 50–1000 with a scan time of 0.03 s and an interscan time of 0.02 s. Leucine enkephalin was used as the lock mass ([M+H]^+^ = 556.2771 in the positive mode and [M−H]^−^ = 554.2615 in the negative mode).

### 2.5. Data Processing and Analysis

All mass spectral data were collected in  .d format and converted to  .mz Data using the Mass Hunter Qualitative Analysis software (Version B 03.01, Agilent Technologies, Palo Alto, CA, USA); XCMS program of R software platform was used for data preprocessing, such as baseline filtering, peak identification, nonlinear retention time alignment, and peak matching. The next data were performed in Excel 2007, and the data were combined to form the final two-dimensional matrix. 958 metabolites in positive mode and 1589 metabolites in negative mode were detected.

To determine distinction between groups, multivariate analyses including principal components analysis (PCA) and partial least squares-discriminate analysis (PLS-DA) [20, 21] were conducted with Simca-P software 11.0 (Umetrics, Umea, Sweden). To obtain more reliable and intuitive results, unit variance (UV) scaling and mean-centered were applied to analyzing the data.

The *R* ^2^ *Y* and *Q* ^2^ values were used to evaluate the model quality, which indicate the fit (*R* ^2^ *Y*) and prediction ability (*Q* ^2^); *R* ^2^ *Y* and *Q* ^2^ values closing to 1.0 means an excellent mathematical model and the values > 0.5 suggest reliable predictive accuracy. Variable importance projection (VIP) constructed from PLS-DA analysis was used to preselect variables (VIP > 1.0 means influential and VIP ≥1.5 means highly influential) and student's* t* test (*P* < 0.05) was selected to measure the significance of each differential metabolite. The metabolites were qualified by the Scripps Center for Metabonomics and Mass Spectrometry (METLIN, http://metlin.Scripps.edu/) and the Human Metabolome Database (HMDB, http://hmdb.ca/); the possible pathways of metabolites were obtained from the Kyoto Encyclopedia of Genes and Genomes (KEGG, http://www.genome.jp/kegg/) and HMDB.

## 3. Results 

### 3.1. Demographic and Clinical Characteristics

Demographic and clinical data of subjects were summarized in Table 2.

### 3.2. Visual Inspection of Chromatography

**Figure 1:**
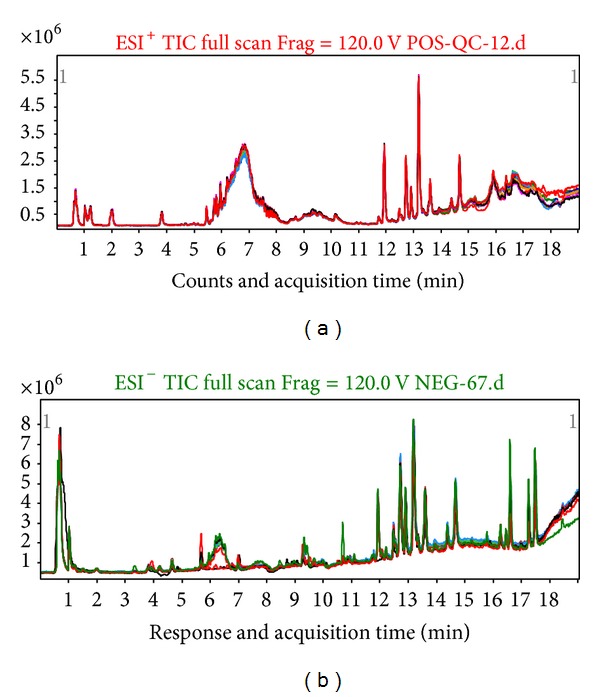
Overlapping total ion current (TIC) chromatograms of plasma samples in (a) positive mode and (b) negative mode.

In order to inspect the performance of LC-MS instrumentation, three blood samples were selected randomly in the positive and negative mode, respectively, for visual inspection; overlapping total ion current (TIC) chromatograms were shown in Figure 1. There appeared to be strong instrumental analysis signal, highlighting peaks capacity and good retention time reproducibility.

### 3.3. Metabolic Profiles of CHD Patients Compared to Healthy Subjects and Detection of Potential Metabolic Biomarkers

**Figure 2:**
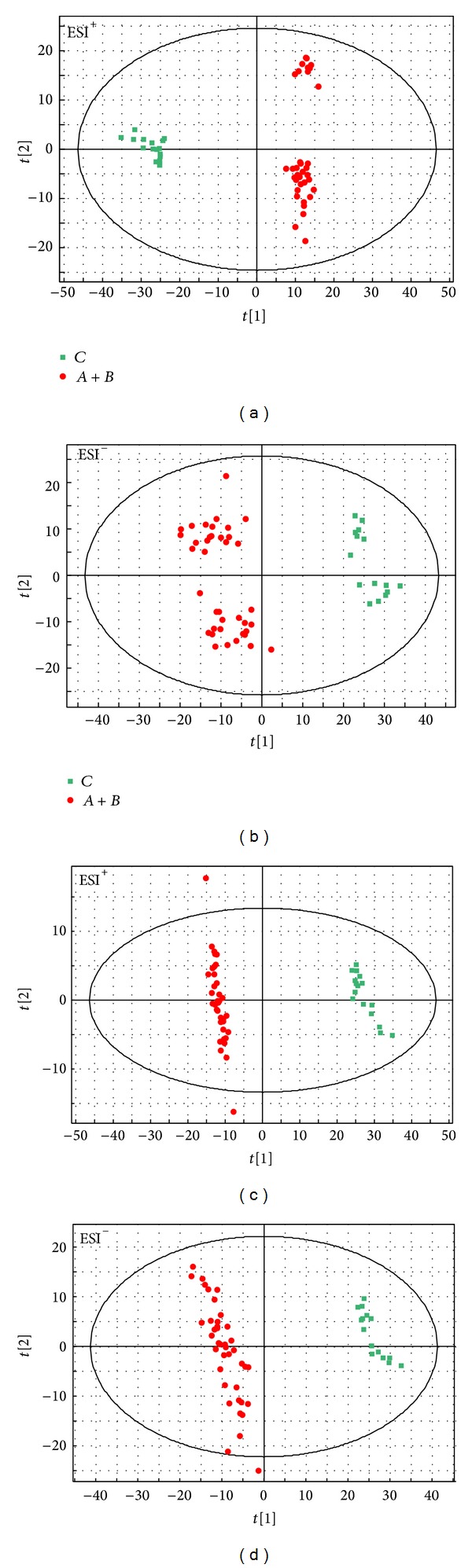
PCA scores plots of CHD (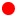) and healthy (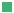) subjects in ESI^+^ (a) mode and ESI^−^ (b), PLS-DA scores plots of CHD (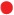) and healthy (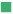) subjects in ESI^+^ (c) mode and ESI^−^ (d).* X*-axis expressed the first component of the metabolite data.* Y*-axis expressed the second component.

The PCA scores plots showed significant discrimination between the CHD group and the healthy control group in both positive (Figure 2(a)) and negative modes (Figure 2(b)). In the score plot, the confidence interval was defined by the Hotelling *T* ^2^ ellipse (95% confidence interval). As exhibited in Figure 2, most samples were within the 95% confidence interval. PLS-DA models confirmed the clear separation tendency between the two groups. The classification results were shown in Figures 2(c) and 2(d). All the models parameters were presented in Table 3.

VIP (from PLS-DA) > 1.5 and *P* value (from student's* t* test) < 0.05 were used to confirm the differential metabolites. Retention time (RT), mass-to-charge ratio (*m*/*z*), differential metabolites, VIP value, *P* value, and fold (changed trend of CHD patients compared to the healthy group) were presented in Table 4 simultaneously. The concentrations of 3 metabolites were significantly upregulated (compared to the healthy controls) in the CHD patients, while the remaining 23 metabolites were significantly downregulated.

### 3.4. ZHENG Differentiation

**Figure 3:**
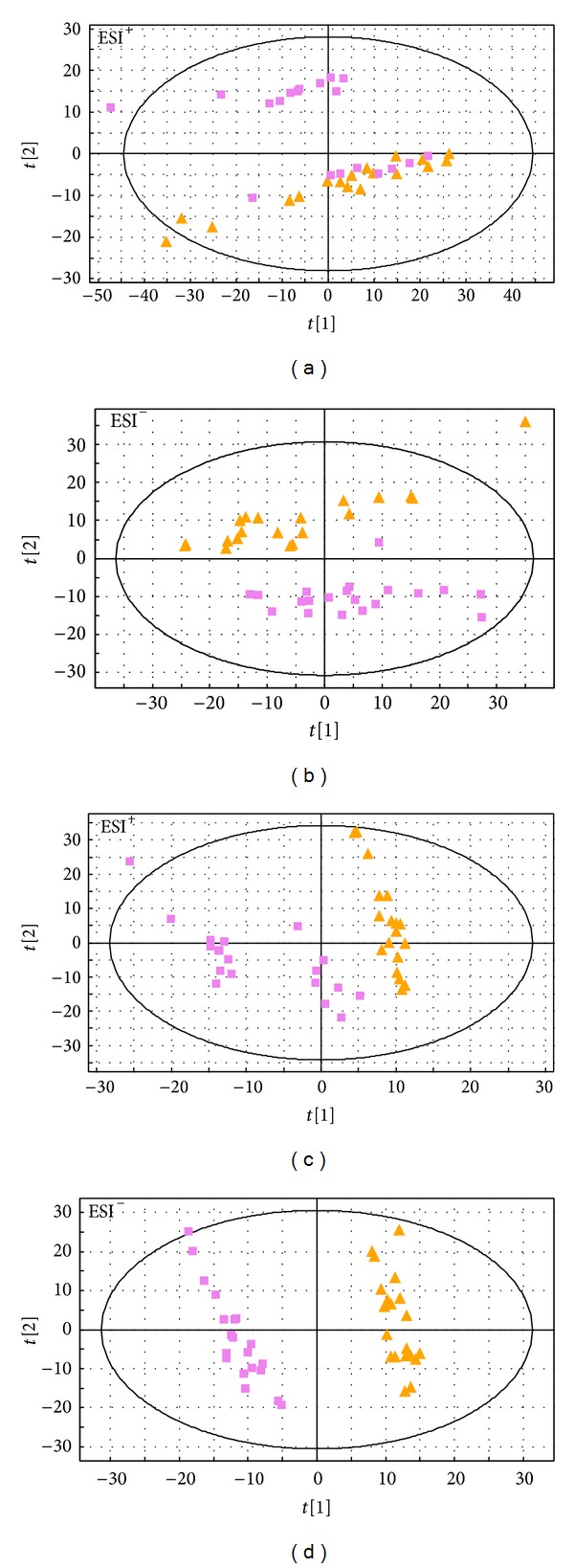
PCA scores plots of CHD with phlegm syndrome (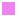) and CHD with blood-stasis syndrome (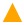) in ESI^+^ (a) mode and ESI^−^ (b), PLS-DA scores plots of the two ZHENG types in ESI^+^ (c) mode and ESI^−^ (d).* X*-axis expressed the first component of the metabolite data.* Y*-axis expressed the second component.

The corresponding models scores plots of the ZHENG types and the parameters were exhibited in Figure 3 and Table 2, respectively. As shown in Figure 3 most samples were within the scope Hotelling *T* ^2^ ellipse. PLS-DA models in both ESI^+^ (Figure 3(c)) and ESI^−^ (Figure 3(d)) modes and PCA model in ESI^−^ (Figure 3(b)) mode illustrated that there was satisfactory classification between phlegm syndrome and blood-stasis syndrome, which reflected the pathological variation between the two ZHENG types.

The differential metabolites contributing to the clustering and discrimination (VIP > 1.5; *P* < 0.05) between phlegm syndrome and blood-stasis syndrome were presented in Table 5; there were 7 metabolites' concentrations in phlegm syndrome significantly higher than that in blood-stasis syndrome while the remaining 11 metabolites were contrary.

## 4. Discussion

Metabonomics profiling study on CHD patients has been investigated many times. For instance, Brindle et al. have reported for the first time that metabonomics analysis of serum samples based on ^1^H-NMR can provide a clinically useful diagnosis of CHD and can be used as a replacement of angiography. Metabolic profiling of biological specimens could be of great significance for clinicians and biologists in the early diagnosis [9]. Sun et al. find 16 differential metabolites between unstable angina (UA) and atherosclerosis patients, which are closely associated with phospholipid metabolism, tryptophan metabolism, and MG metabolism. These potential biomarkers can provide information on understanding the biological mechanisms of UA and the diagnosis of UA [22]. In our previous studies with GC-MS technology, we obtained 32 potential biomarkers of CHD patients, including amino acids, fatty acids, organic acids, glucose, and alcohol esters [23].

**Figure 4:**
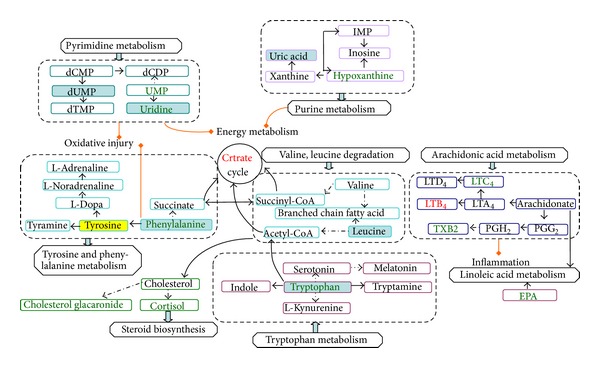
Disturbed metabolic pathways. Green font denoted biomarkers downregulations and red font denoted biomarkers upregulations (CHD/healthy people); yellow area denoted metabolites higher level and blue area denoted lower level (phlegm/blood stasis in CHD).

In the research, 26 potential biomarkers that discriminated CHD patients from healthy controls were obtained. We found that the potential biomarkers were principally involved in arachidonic acid metabolism, amino acid metabolism, purine metabolism, pyrimidine metabolism, steroid biosynthesis, and linoleic acid metabolism, which were mainly correlated to inflammation, amino acid dysfunction, energy metabolism dysfunction, and oxidative injury in the pathological development of CHD. The disturbed metabolic pathway was shown in Figure 4.

(1) Inflammation: four biomarkers including TXB1, TXB2, leukotriene C4, and Leukotriene B4 belong to the pathway of arachidonic acid metabolism which could cause inflammation and lipid infiltration [24]. The leukotrienes have been identified as primarily proinflammatory mediators. LTB4 can activate leukocytes and promote conversion of monocyte into foam cells. Increased leukocytes adsorb to vascular endothelium [25]; foam cells accumulate and form fatty streak and lipid plague. LTC4 increase permeability of vascular in postcapillary venules and lead to potent vasoconstrictive effects on coronary arteries [26]. The result supported that activation of inflammation plays a role in the pathogenesis of CHD.

(2) Amino acid dysfunction: concentrations of tryptophan and phenylalanine were lower in the plasma of the CHD group than the healthy group which indicated that the amino acid metabolic pathways were disrupted in CHD patients. Tryptophan is considered an important mechanism in immune responses. Tryptophan catabolism suppresses helper T cell (Th1) activity [27]; the latter can activate macrophages, contribute to the activation of inflammation like delayed hypersensitivity [28, 29], and promote the formation of coronary atherosclerotic plaque. Consequently, degradation of tryptophan reflects an anti-inflammatory pathway and counterregulatory protective mechanism on early atherosclerotic lesions. However, permanent state of immune activation and low level of tryptophan may contribute to development of immunodeficiency [30]. Downregulation of tryptophan has been demonstrated in other studies about CHD [31, 32], and similar relationships between diminished tryptophan and immune activation are found in gynecological cancer, sepsis [33], rheumatoid arthritis [34], and Parkinson's disease [35]. Another pathway of tryptophan is the generation of the neurotransmitter 5-hydroxytryptamine (5-HT), which is a strong modulator of emotional behavior in the central nervous system. Decreased tryptophan induces declined 5-HT which was possibly responsible for depressive mode and sleep disorder of CHD patients.

Phenylalanine is metabolised to tyrosine, and the latter is decarboxylated to tyramine and also to Dopa, dopamine, and noradrenaline, as shown in Figure 4. Lower level of phenylalanine was consistent with previous study [36, 37] which is possibly related to neuropsychiatric disturbances [38].

(3) Energy metabolism dysfunction: there were two pathways including purine metabolism (hypoxanthine, 2-aminoadenosine) and pyrimidine metabolism (uridine, uridine monophosphate) that contributed to energy imbalance in CHD patients. When myocardial energy metabolism dysfunction occurs owing to ischemia, the nucleotide degradation pathway is activated. In fact, the relevance between myocardial ATP exhaustion and the increased hypoxanthine and uridine has been clearly observed [39]; in addition, uridine has been considered a marker of myocardial viability after coronary occlusion and reperfusion and an indirect indicator of tissue energy crisis [40]. Increased uridine and decreased hypoxanthine were found in previous metabonomics study of myocardial infarction in rats [31, 41]; however, both hypoxanthine and uridine downregulated in our study and drug medication possibly play a role. On the other hand, when hypoxanthine converts to xanthine, reactive oxygen species (ROS) generate which is the characteristic of hypoxia and play a major role in microvascular dysfunction and exert direct tissue damage [42]. Citric acid cycle obstacle result in the accumulation of citric acid in the plasma, oxalosuccinic acid belongs to the citric acid cycle, and increased oxalosuccinic acid is found in a study [31] which is kept in step with increased citric acid in our study. Multiple metabolites in a particular pathway correlating with the perturbation will be found in a further study which help us understand the mechanisms of CHD better.

(4) Other metabolic abnormalities: the concentrations of cortolone-3-glucuronide and cortisol involved in steroid metabolism and cholesterol glucuronide involved in cholesterol were lower in CHD patients in our study.

We noticed that there is no significant difference in lipid metabolites between CHD patients and healthy people which would be interpreted with the intervention of lipid-lowering medication. Drugs could confound the metabonomics data; however, we still found clear separate trend between the groups. We will select more cases to validate the result in a further study.

As shown in Figure 4, there were 18 differential metabolites mainly involved in amino acid metabolism (leucine, phenylalanine, tryptophan, and tyrosine), purine metabolism (uric acid, ubiquinone), and pyrimidine metabolism (dUMP, uridine), which exhibited a satisfactory classification between phlegm syndrome and blood-stasis syndrome in CHD patients. The levels of amino acid (except phenylalanine) were lower in phlegm syndrome than in blood-stasis syndrome of CHD, which indicated different degrees of disorder in amino acid metabolism of the two ZHENG types. However, some study found that severity of the amino acid metabolic disorders in phlegm syndrome is similar to blood-stasis syndrome of CHD [43], the difference being possibly caused by using different metabonomics technology and analysis method. Increased phenylalanine to tyrosine ratio (Phe/Tyr) represents immune activation and higher Phe/Tyr was found in blood-stasis syndrome of CHD group.

Uridine, uric acid, and dUMP were increased in CHD patients with blood stasis compared to phlegm syndrome; uridine and uric acid were the main nucleotide catabolite of myocardial ischemia and the level of uric acid in plasma is significantly relevant to risk of cardiovascular atherosclerotic disease and cardiovascular disease mortality [38, 44–47]. Uric acid also contributed to oxidative injury [48]. dUMP was involved in oxidative injury and uridine related to energy metabolism.

The differences in inflammation, oxidative injury, and energy metabolism reflected phlegm syndrome and blood-stasis syndrome in CHD belonging to different metabolism patterns; in addition, they were possibly different stages of the CHD pathogenesis development [49]. Biomarkers confirmed in the further study will enhance the ability to identify ZHENG types of phlegm and blood stasis in CHD and reduce the artificial error in identifying syndrome differentiation. It could provide widespread population screening and ZHENG types standardization in TCM.

As a preliminary study, we considered that LC-MS-based metabonomics can be useful to study the underlying essence of the disease (CHD) and ZHENG types (phlegm and blood syndrome). However, the sample size was small and the case-control design limits causality in this study; more subjects will be needed to confirm the potential biomarkers in subsequent research; meanwhile, we will use another technique, H^1^-NMR, to validate the separated trend and acquire more potential biomarkers. In addition, the extraneous variables, such as age, gender, smoking habits, complications (such as hypertension, diabetes), and treatment will be stratified to improve reliability of these potential biomarkers. Furthermore, validation with other study populations will be needed to identify reliability of the models in the future study.

## 5. Conclusions

In our study, CHD subjects were distinguished from the healthy volunteers utilizing the method of metabonomics based on LC-Q-TOF/MS and multivariate statistical analysis (PCA and PLS-DA). Differential metabolites between CHD patients and healthy subjects were mainly involved in arachidonic acid metabolism, amino acid metabolism, purine metabolism, pyrimidine metabolism, and steroid biosynthesis, which were related to inflammation, amino acid dysfunction, energy metabolism dysfunction, and oxidative injury. The two ZHENG types (phlegm and blood stasis) in this study were also obviously discriminated by metabonomics approach. The significant difference in concentration of metabolites demonstrated that they were probably different stages of the CHD pathogenesis development.

## Supplementary Material

The Supplementary Material Depicted the Clinical Data and Treatment of Each Coronary Heart Disease Patients and Healthy Control.

## Figures and Tables

**Table 1:** The gradient program of mobile phase.

Time (min)	Flow rate (mL/min)	A (%)	B (%)
0	0.4	95	5
2	0.4	95	5
17	0.4	5	95
19	0.4	5	95

**Table 2:** Demographic and clinical characteristics of subjects.

Characteristics	Blood stasis syndrome *n* = 20	Phlegm syndrome *n* = 20	Healthy *n* = 16
Age (years)	61.05 ± 11.77	62.08 ± 10.15	60.27 ± 12.04
Male	13 (65%)	17 (85%)	10 (62.5%)
BMI (kg/m^2^)	23.11 ± 1.45	27.72 ± 0.86	23.60 ± 0.87
Hypertension	6 (30%)	10 (50%)	6 (37.5%)
TG (mmol/L)	1.63 ± 0.90	1.54 ± 1.02	1.23 ± 0.77
TC (mmol/L)	4.15 ± 0.98	4.11 ± 0.93	3.25 ± 0.72
LDL (mmol/L)	2.21 ± 0.93	2.08 ± 0.93	2.02 ± 0.44
HDL (mmol/L)	1.19 ± 0.24	1.14 ± 0.48	1.27 ± 0.28
Apo (a_1_) (g/L)	1.29 ± 0.21	1.17 ± 0.21	1.33 ± 0.14
Apo (b) (g/L)	1.04 ± 0.33	0.77 ± 0.19	0.62 ± 0.30
LDH (U/L)	225.78 ± 137.61	206.31 ± 56.52	158.01 ± 42.21
CK (U/L)	253.96 ± 691.83	81.39 ± 45.08	73.30 ± 11.68
CK-MB (U/L)	19.27 ± 21.95	19.11 ± 10.02	24.45 ± 14.80
Myoglobin (ug/L)	41.80 ± 29.09	43.28 ± 22.07	32.40 ± 10.67
CTn1 (ug/L)	0.12 ± 0.21	0.44 ± 0.29	0.17 ± 0.11

**Table 3:** Summary of the parameters for modeling quality.

Group	Mode	PCA model			PLS-DA model			
		^ e^No	*R* ^2^ *X*	*Q* ^2^ *Y*	^ e^No	*R* ^2^ *X*	*R* ^2^ *Y*	*Q* ^2^ *Y*
CHD/healthy	ESI^+^	2	0.433	0.378	2	0.376	0.993	0.981
CHD/healthy	ESI^−^	3	0.349	0.187	3	0.237	0.989	0.969
A/B	ESI^+^	2	0.430	0.349	4	0.508	0.989	0.913
A/B	ESI^−^	3	0.279	0.105	3	0.253	0.994	0.928

A represented phlegm syndrome in CHD, B represented blood-stasis syndrome in CHD, and ^e^No represented amount of components.

**Table 4:** Plasma metabolites for discriminating CHD patients from healthy controls.

Mode	RT	Mass	Metabolite	VIP	*P*	^ a^Fold
EIS^+^	6.21	542.28	Cortolone-3-glucuronide	1.75	7.02*E* − 44	−25.59
	5.74	431.21	17-Phenoxy trinor PGF2*α* ethyl amide	1.74	1.59*E* − 37	−25.32
	5.71	562.37	Cholesterol glucuronide	1.74	1.34*E* − 35	−25.35
	5.03	362.22	Cortisol	1.73	7.10*E* − 34	−24.65
	7.52	890.51	Dipalmitoyl phosphatidylinositol 3-phosphate	1.73	1.28*E* − 33	−25.53
	15.91	370.24	TXB2	1.73	3.64*E* − 31	−24.54
	13.41	393.3	PGH2-EA	1.71	2.30*E* − 28	−25.08
	15.90	298.18	13,14-Dihydro-15-keto-tetranor PGE2	1.70	5.56*E* − 27	−25.84
	15.90	372.25	TXB1	1.70	1.42*E* − 26	−26.76
	11.98	338.17	18-Carboxy dinor leukotriene B4	1.70	1.55*E* − 26	−24.83
	8.98	413.26	15-Keto-17-phenyl trinor Prostaglandin F2*α* ethyl amide	1.66	8.12*E* − 22	−24.57
	14.52	510.28	Leukotriene D4 methyl ester	1.65	8.28*E* − 21	−22.56
	6.71	427.27	17-Phenyl trinor prostaglandin F2*α* Cyclopropyl amide	1.61	1.41*E* − 18	−24.27
	16.05	348.23	PGA2 methyl ester	1.59	2.34*E* − 17	−22.23
	5.77	472.24	Chenodeoxycholic acid 3-sulfate	1.50	1.24*E* − 13	−23.70

EIS^−^	1.03	244.07	Uridine	1.98	7.81*E* − 12	−0.72
	16.61	348.23	PGA2 methyl ester	1.98	9.09*E* − 12	3.52
	1.02	136.04	Hypoxanthine	1.90	1.66*E* − 10	−1.84
	1.03	192.03	Citric acid	1.89	2.40*E* − 10	2.12
	3.81	204.09	L-Tryptophan	1.88	3.85*E* − 10	−1.01
	3.81	324.03	Uridine monophosphate (UMP)	1.81	3.09*E* − 09	−1.98
	2.00	165.08	L-Phenylalanine	1.61	5.73*E* − 07	−1.30
	4.66	282.11	2-Aminoadenosine	1.56	1.57*E* − 06	−2.41
	13.16	625.30	Leukotriene C4	1.55	1.83*E* − 06	−2.20
	16.62	302.22	EPA	1.55	1.87*E* − 06	−2.47
	16.88	336.23	LTB4	1.53	2.68*E* − 06	4.50
	1.91	116.05	2-Keto valeric acid	1.51	4.25*E* − 06	−2.05

^a^Fold = log_2_(average peak intensity of CHD group/average peak intensity of healthy group), “−” represented downregulated compared to healthy group, and “+” represented upregulated compared to healthy group.

**Table 5:** Plasma differential metabolites for discriminating phlegm from blood-stasis syndrome.

Mode	RT	Mass	Metabolite	VIP	*P*	^ a^Fold
EIS^+^	1.21	131.10	L-Leucine	1.83	9.54*E* − 04	−0.62
	1.02	168.03	Uric acid	1.73	1.91*E* − 03	−1.32
	2.00	165.08	L-Phenylalanine	1.68	2.81*E* − 03	−0.37
	3.82	204.09	L-Tryptophan	1.62	4.05*E* − 03	−0.26
	10.94	250.12	Ubiquinone	1.57	5.38*E* − 03	−0.81
	15.21	384.29	17,20-dimethyl prostaglandin F1*α*	1.57	5.61*E* − 03	0.75
	3.82	145.05	Isoquinoline N-oxide	1.55	6.08*E* − 03	−0.26
	10.96	393.29	PGH_2_-EA	1.54	6.46*E* − 03	0.43
	17.48	283.29	Stearamide	1.54	6.56*E* − 03	−1.71
	11.74	568.33	Deoxycholic acid 3-glucuronide	1.52	7.62*E* − 03	0.88
	16.85	368.29	Octadecyl fumarate	1.50	8.10*E* − 03	1.10

EIS^−^	10.28	250.12	Ubiquinone	2.70	5.97*E* − 10	−1.15
	17.23	363.24	N-Palmitoyl taurine	2.58	1.05*E* − 08	−2.33
	17.46	389.26	N-Oleoyl taurine	2.57	1.11*E* − 08	−1.95
	16.68	552.19	Deoxycholic acid disulfate	2.26	2.43*E* − 06	−2.08
	1.08	181.07	L-Tyrosine	1.76	5.73*E* − 04	0.56
	1.03	244.07	Uridine	1.70	9.09*E* − 04	−0.28
	0.74	130.03	Itaconic acid	1.68	1.06*E* − 03	1.44
	1.04	308.04	Deoxyuridine monophosphate (dUMP)	1.63	1.64*E* − 03	−1.04
	16.75	330.26	Eicosapentaenoic acid ethyl ester	1.63	1.65*E* − 03	4.77

^a^Fold = log_2_(average peak intensity of phlegm syndrome/average peak intensity of blood stasis syndrome); “−” represented downregulated compared to blood stasis syndrome; “+” represented upregulated compared to blood stasis syndrome.
